# Endophytic Isolates of Cordyceps fumosorosea to Enhance the Growth of Solanum melongena and Reduce the Survival of Whitefly (Bemisia tabaci)

**DOI:** 10.3390/insects11020078

**Published:** 2020-01-22

**Authors:** Tingfei Sun, Zhang Shen, Mobeen Shaukat, Cailian Du, Shaukat Ali

**Affiliations:** 1 Key Laboratory of Bio-Pesticide Innovation and Application, Guangzhou 510642, China; suntingfei@stu.scau.edu.cn (T.S.); aliscau@gmail.com (M.S.); Ducailian@stu.scau.edu.cn (C.D.); 2 Engineering Research Center of Biological Control, Ministry of Education and Guangdong Province, South China Agricultural University, Guangzhou 510642, China; 3 Shaoyang Tobacco Company, Shaoyang 422100, China

**Keywords:** *Cordyceps fumosorosea*, *Solanum melongena*, *Bemisia tabaci*, *entomopathogenic fungi*, endophytic isolates

## Abstract

This study reports the effects of seed treatment with *Cordyceps fumosorosea* on seed germination, growth, colonization of eggplant (*Solanum melongena*), and growth of *Bemisia tabaci* (feeding on fungal colonized eggplant leaves). Germination rates of eggplant seeds were similar among different treatments. The growth parameters such as root length, shoot length, and number of leaves) differed significantly after 15, 30, and 60 days of seed treatment. The total dry weight of eggplant in response to treatment with *C. fumosorosea* isolates increased significantly when compared with the control. Both isolates of *C. fumosorosea* colonized different plant tissues, although the extent of colonization decreased during the experimental period. The colonization of eggplants by both *C. fumosorosea* isolates resulted in a significant reduction of B. *tabaci* incidence. This study possibly provides the first report of increased plant growth and increased insect mortality in eggplants inoculated with *C. fumosorosea* isolates.

## 1. Introduction

The development of sustainable strategies to improve crop protection is a major challenge facing agricultural scientists [1]. The use of entomopathogens for pest control can provide an environmentally friendly alternative to chemical pesticides [2]. Several species of *entomopathogenic fungi* are being used or developed as biopesticides against different insect pests [3]. *Entomopathogenic fungi* are normally applied in spray formulations as pest control agents for a short period of time [4]. Recent advances in research have shown that *entomopathogenic fungi* can play a role in plant–microbial symbioses by which they can protect plants against different insects and diseases [5]. Furthermore, there is growing evidence that *entomopathogenic fungi* enhance plant growth by improved nutrient uptake, hormone production, and tolerance to different abiotic as well as biotic stresses [2,6]. These entomopathogenic fungal endophytes are classified as non-clavicipitaceous because they are usually transmitted horizontally [7]. Different species of fungi are known to enhance plant growth as well reduce insect pest growth when colonized into plants [2,8,9,10]. For example, *B. bassiana* acted as an endophyte to wheat seedlings by increasing plant spike production and effectively controlling *Spodoptera litura* larvae [2].

*Bemisia tabaci* (Gennadius) (Homoptera: Aleyrodidae), also known as sweet potato whitefly, is a major crop pest worldwide [11]. Furthermore, *B. tabaci* acts as a vector of around 150 plant viruses [12]. In China, MEAM1 *B. tabaci* is well distributed across 31 provinces or municipalities where it causes huge economic crop losses [13]. *Bemisia tabaci* is considered to be one of the most destructive pests of eggplant [14]. Islam et al. [15] showed that *B. tabaci* infestation caused reduction in the leaf area (26.6%), leaf fresh weight (21.8%), and leaf dry weight (19.27%) of eggplant when compared to non-infested plants. Their study also reported a 9.7% reduction in chloprophyll content and a 65.9% reduction in the rate of photosynthesis in response to whitefly infestation when compared with the control. The management of whitefly is mainly carried out by using synthetic chemicals, which is causing harmful effects to our natural environment [16]. Therefore, efforts have been made to find an environmentally safer alternative to synthetic pesticides and the use of *entomopathogenic fungi* can be one of the potential alternatives [17]. The entomopathogenic fungus, *Cordyceps fumosorosea* Zare and Gams (Hyphomycetes) (formerly *Isaria fumosorosea*, designated as *Cordyceps* clade; Kepler et al. [18]), is a well known fungal species being used for whitefly management [19]. Strains of *C. fumosorosea* have also been proven to be pathogenic against various insect species in different regions of the world [20,21,22]. Huang et al. [21] studied the virulence of *C. fumosorosea* against *B. tabaci*. Their results indicate that strain PF01-N4 is virulent against *B. tabaci,* having a LC_50_ value of 2.16 × 10^6^ and 1.10 × 10^4^ conidiamL^−1^ after 6 and 12 days, respectively. Since the 1990s commercial preparations of *C. fumosorosea* have been used against whiteflies infesting different crops [23,24]. *Cordyceps fumosorosea* has received much attention because of its microbial potential, but this fungus can also show endophytic behavior [25]. To date, a few studies have been carried out to observe the endophytic behaviour of *C. fumosorosea* and its subsequent effects on plant growth as well as pest management. Therefore, this study was performed (a) to assess the effects of *C. fumosorosea* colonization on plant growth, and (b) to observe the influence of *C. fumosorosea* on the mortality of *B. tabaci* individuals feeding on eggplants.

## 2. Materials and Methods

### 2.1. Host Plant

Eggplant (*Solanum melongena*) was opted for this study due to its ability of adapting different climates, faster growth, and its dietary value. Seeds of eggplant variety, “Dafeng”, obtained from Guangdong Academy of Agricultural Sciences, Guangzhou, P.R. China were surface sterilized by following the method of Jaber and Enkerli [8]. Briefly, seeds were soaked in sodium hypochlorite (NaOCl) solution (1%) for two minutes, followed by soaking in 70% ethanol for two minutes. The seeds were then rinsed thrice with sterile distilled water. The effectiveness of sterilization was confirmed through plating the sterilized seeds on potato dextrose agar (PDA) for 7 days in a biological incubator (26 ± 2 °C, 0:24 h L:D).

### 2.2. Fungal Inoculum

*Cordyceps fumosorosea* isolates SP502 and SP535 originally isolated from soil, obtained from the repository of the Key Laboratory of Biopesticides Innovation and Application of Guangdong Province, South China Agricultural University, Guangzhou, were used for the studies. The culturing, as well as basal concentration (1 × 10^7^ conidia ml^−1^) preparation of both isolates, was performed by following the method of Ali et al. [26].

### 2.3. Effect of Fungal Inoculation on Germination of Seeds

Surface sterilized eggplant seeds (10 seeds) were soaked in fungal suspension (1 × 10^7^ conidia ml^−1^ prepared in ddH_2_O having 0.1% Tween 80) of both isolates for 8 h. Seeds soaked in sterile ddH_2_O containing 0.1% Tween 80 served as control. The flasks containing seeds were placed in the dark at 25 ± 2 °C. After soaking, the seeds were moved to Petri dishes (9 cm ø) with moist filter paper. The filter paper was kept moist by the addition of sterile ddH_2_O at regular intervals. The Petri dishes were placed in the dark at 25 ± 2 °C. The whole experimental setup was repeated five times (n = 5) and a total of 50 seeds were used for each treatment. The number of germinated seeds was recorded on a daily basis.

### 2.4. Effect of Fungal Inoculation on Plant Growth

The effects of *C. fumosorosea* endophytic colonization on plant growth parameters were observed by planting the inoculated or control seeds (soaked in fungal suspension or distilled water, as described above) in disinfected pots. The planting substrate was prepared by following Jaber and Enkerli [8]. Seeds (2 seeds per pot) were sown for each treatment and ten pots were used per treatment. The whole experimental setup was repeated five times (n = 5). Potted plants were placed at 25 ± 2 °C, 60 ± 5% R.H, and 16 h L: 8 h D photoperiod. Plants were watered as needed throughout the experiment for 60 days. Five seedlings were carefully and randomly removed from the pots after 15, 30, and 60 days of fungal inoculation and roots were washed clean with tap water. Different plant parts (roots, shoots and leaves) were separated and the lengths of roots and shoots, as well as the number of leaves were measured. Different plant parts (roots, shoots and leaves) were then held at 70 °C until becoming constant dry weights.

### 2.5. Endophytic Colonization of Eggplants by C. fumosorosea.

The systemic colonization of eggplants by *C. fumosorosea* was observed by re-isolating the fungi from different plant parts at 30 days, 45, and 60 days post inoculation by following the method of Arnold et al. [27]. The plants used to observe the growth parameters were later used for re-isolation experiments. Plant roots, shoots, and leaves were separated, followed by surface sterilization with 0.5% NaOCl (for leaf and shoot tissues) or 1% NaOCl (for roots). The tissues were washed with 70% ethanol for 2 min, followed by three rinses of ddH_2_O. Plant materials were then air dried in laminar flow hood to remove the water droplets. Eight pieces of each of the different plant parts were prepared for inoculation on PDA plates by using the method described by Jaber and Enkerli [8]. Plates were placed at 25 ± 2 °C and fungal growth was recorded on a daily basis for 2 weeks. The whole experiment was repeated five times (n = 5). The colonization of different plant parts (%) by both *C. fumosorosea* isolates was calculated through the equation by Petrini and Fisher [28], as shown below;

$$Percent\;colonization=(\frac{number\;of\;plant\;tissues\;showing\;fungal\;growth}{Total\;number\;of\;plant\;tissues\;plated})\times 100\%$$

### 2.6. Effect of C. fumosorosea Colonization on Growth of Bemisia tabaci

The *Bemisia tabaci* population, obtained from the stock cultures of Key Laboratory of Biopesticides Innovation and Application of Guangdong Province, South China Agricultural University, Guangzhou, was maintained on eggplants in cages placed under laboratory conditions at 25 ± 2 °C, 70 ± 5% R.H, and a photoperiod of 12 h L: 12 h D by following Ali et al. [29]. Newly emerged adults were directly collected from the cages and were used for the experiment.

Eggplants inoculated with both *C. fumosorosea* isolates or uninoculated plants were moved to bioassay cages (60 cm × 60 cm × 60 cm) by following Zhang et al. [30]. Micro-cages were attached to the under surface of eggplants inoculated with *C. fumosorosea* isolates 30 days post inoculation. Two pairs of whitefly adults (each pair consisting of one male and one female) were released in each cage for oviposition. The micro-cages were removed after 24 h and the number of eggs was adjusted to 10 eggs per leaf on 5 different leaves making up a total of 50 eggs per plant. In the control, eggs were inoculated on eggplant leaves without fungal inoculation. The whole experimental setup was repeated five times (n = 5). The leaves bearing eggs were monitored for the number of eggs hatched and the newly hatched *B. tabaci* immatures were circled for close monitoring by following Zhang et al. [31]. The survivorship of *B. tabaci* immature (nymphs and pupae) was recorded on a daily basis for 30 days and reduction in *B. tabaci* population was calculated by following Zhang et al. [31]. To survey the infective mortalities, cadavers were taken out and cultured separately at 25 ± 2 °C and >95% R.H. to stimulate sporulation. If conidia spores of *C. fumosorosea* were recovered from a cadaver, the cadaver was regarded as having died from infection by *C. fumosorosea.*

### 2.7. Data Analysis

The data regarding plant growth parameters, fungal colonization and reduction in whitefly population were subjected to one-way analysis of variance (ANOVA-1) at a 5% level of significance. Treatment means were compared by Tukey’s HSD test. All the statistical analyses were performed using SAS ver 9.1 [32].

## 3. Results

### 3.1. Effect of Fungal Inoculation on Seed Germination

**Figure 1:**
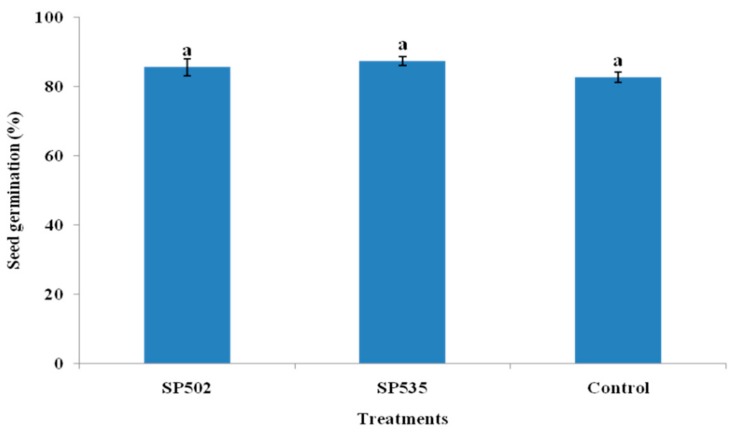
Effect of *Cordyceps fumosorosea* isolates on percent germination of eggplant seeds. Error bars indicate the standard error of means based on five replicates (n = 5). Bars having different letters indicate that means are significantly different from each other at a 5% level of significance.

Germination of eggplant seeds soaked in conidial suspension of *C. fumosorosea* isolates and control were statistically similar to each other (F_2,12_ = 21.84; *p* = 0.056) (Figure 1).

### 3.2. Effect of Fungal Inoculation on Plant Growth Parameters

**Figure 2:**
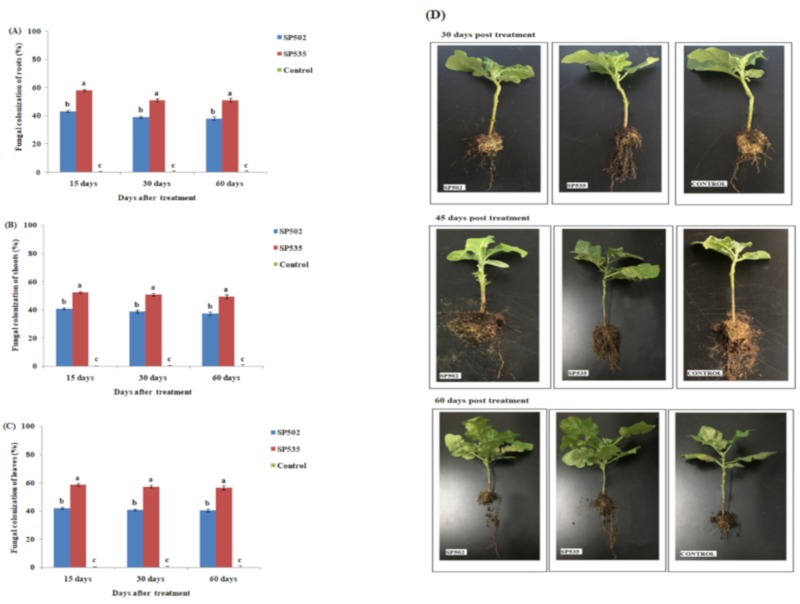
Effect of *Cordyceps fumosorosea* isolates on growth parameters of eggplant after 30, 45, and 60 days of fungal treatment. (**A**) Root length; (**B**) shoot length; and (**C**,**D**) number of leaves. Error bars indicate the standard error of means based on five replicates (n = 5). Bars having different letters indicate that means are significantly different from each other at 5% level of significance.

**Figure 3:**
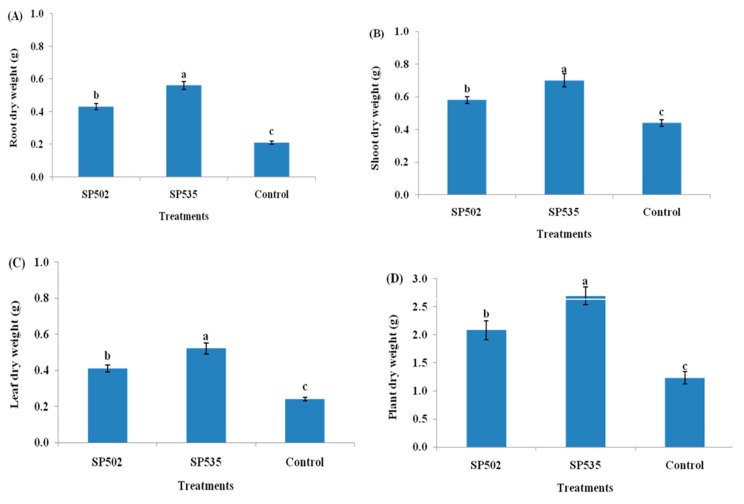
Effect of *Cordyceps fumosorosea* isolates on dry weights of different plant parts after 60 days of fungal treatment. (**A**) Root dry weight; (**B**) shoot dry weight; (**C**) leaf dry weight; and (**D**) total plant dry weight. Error bars indicate the standard error of means based on five replicates (n = 5). Bars having different letters indicate that means are significantly different from each other at 5% level of significance.

The plant growth parameters (root length, shoot length, number of fresh leaves, plant dry weight, root dry weight, shoot dry weight, and leaf dry weight) following the colonization of *C. fumosorosea* isolates showed significantly higher values after 30 (F_2,12_ =3 2.92; *p* < 0.01), 45 (F_2,12_ = 41.82; *p* < 0.01), and 60 days (F_2,12_ = 28.61; *p* < 0.01) of fungal treatment when compared to control (Figure 2 and Figure 3). The *C. fumosorosea* isolate SP535 proved to be a better inducer of plant growth when compared with *C. fumosorosea* isolate SP502 and control (Figure 2 and Figure 3). The root growth in response to *C. fumosorosea* SP535 treatment was 68%, 110%, and 147% higher than control after 30, 45, and 60 days of fungal treatment, respectively (Figure 2A). The shoot growth in response to *C. fumosorosea* SP535 treatment was 109%, 127%, and 103% higher than control after 30, 45, days and 60 days of fungal treatment, respectively (Figure 2B). The number of plant leaves in response to *C. fumosorosea* SP535 treatment were 100%, 125%, and 180% higher than control after 30, 45, and 60 days of fungal treatment, respectively (Figure 2C).

The *C. fumosorosea* SP535 treatment resulted in 118% higher plant dry weight compared with control, whereas plant dry weight in response to *C. fumosorosea* SP502 was 69% higher than the control (Figure 3D). *Cordyceps fumosorosea* SP535 treatment caused 166% higher root dry weight production compared with control, whereas root dry weight in response to *C. fumosorosea*SP502 was 104% higher than control (Figure 3A). *Cordyceps fumosorosea* SP535 treatment resulted in 59% higher shoot dry weight compared with control, whereas shoot dry weight in response to *C. fumosorosea*SP502 was 31% higher than control (Figure 3B). *Cordyceps fumosorosea*SP535 treatment resulted in 116% higher leaf dry weight compared with control, whereas leaf dry weight in response to *C. fumosorosea* SP502 was 71% higher than control (Figure 3C).

### 3.3. Endophytic colonization of eggplant by C. fumosorosea.

**Figure 4:**
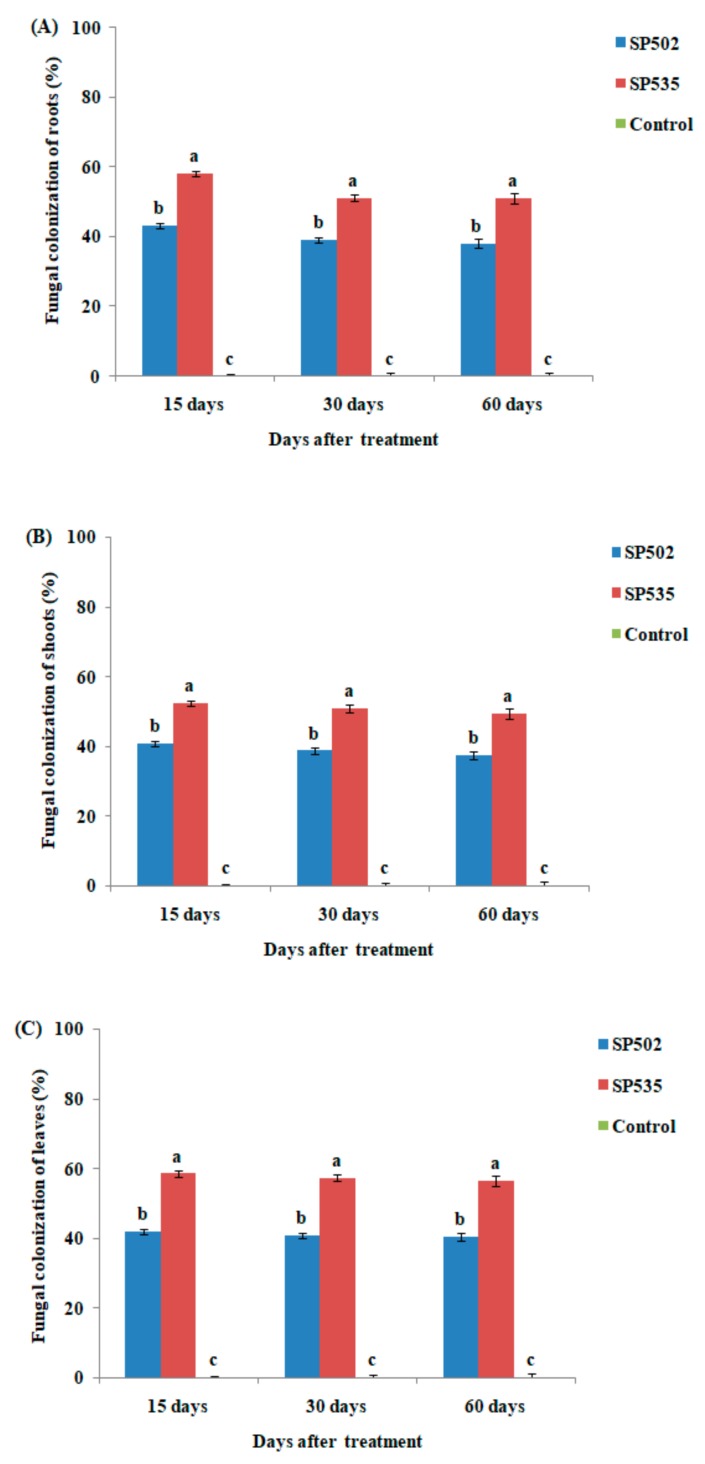
Percent colonization of different plant parts by *Cordyceps fumosorosea* isolates after 30 days, 45 days and 60 days of fungal treatment. (**A**) Roots; (**B**) shoot; and (**C**) leaves. Error bars indicate the standard error of means based on five replicates (n = 5). Bars having different letters indicate that means are significantly different from each other at 5% level of significance.

The effect of fungal inoculation on percent colonization (%) of different plant regions (root, stem and leaf) by *C. fumosorosea* after 30 (F_2,12_ = 18.54; *p* < 0.01), 45 (F_2,12_ = 22.39; *p* < 0.01), and 60 days (F_2,12_ = 31.82; *p* < 0.01) of inoculation has been shown in Figure 4. The fungal colonization of eggplant roots varied significantly between both *C. fumosorosea* isolates after 30, 45, and 60 days of fungal treatment. *Cordyceps fumosorosea* SP535 treatment resulted in 58%, 51%, and 52% colonization of roots after 30, 45, and 60 days of fungal treatment, respectively whereas 43%, 39%, 38% of plant roots were colonized after 30, 45, and 60 days of treatment with *C. fumosorosea* SP502 (Figure 4A).

The fungal colonization of eggplant shoots varied significantly between different *C. fumosorosea* isolates and control after 30, 45, and 60 days of fungal treatment. *Cordyceps fumosorosea*SP535 treatment resulted in 52%, 50%, and 49% colonization of shoots after 30, 45, and 60 days of fungal treatment, respectively, whereas 40%, 38%, and 37% of plant shoots were colonized after 30, 45, and 60 days of treatment with *C. fumosorosea* SP502 (Figure 4B).

The fungal colonization of eggplant leaves at different time intervals varied significantly among different *C. fumosorosea* isolates and control after 30, 45, and 60 days of fungal treatment. *Cordyceps fumosorosea* SP535 treatment resulted in 58%, 57%, and 56% colonization of leaves after 15, 30, and 60 days of fungal treatment, respectively, whereas 41%, 40%, and 40% of plant leaves were colonized after 30, 45, and 60 days of treatment with *C. fumosorosea* SP502 (Figure 4C).

### 3.4. Effect of C. fumosorosea Colonization on Growth of B. tabaci

The colonization of eggplants by both *C. fumosorosea* isolates significantly affected the egg hatchability (%) of *B. tabaci* when compared with control after 15 and 30 days of insect inoculation (Table 1). The egg hatchabilty (%) of *B. tabaci* in response to plant colonization by *C. fumosorosea* isolate SP535 at the end of experimental period was 73.10 ± 2.64%, whereas the percent hatchability of *B. tabaci* eggs following the colonization of *C. fumosorosea* isolate SP502 was 80.34 ± 3.56% (Table 1). 

The colonization of eggplants by both *C. fumosorosea* isolates caused significantly different mortalities of *B. tabaci* pupa when compared with control after 15 days and 30 days of insect inoculation (Table 2). The percent pupal mortality in response to plant colonization by *C. fumosorosea* isolate SP535 at the end of experimental period was 33.19 ± 2.64%, whereas the percent pupal mortality following the colonization of *C. fumosorosea* isolate SP502 was 28.40 ± 3.56% (Table 2).

**Figure 5:**
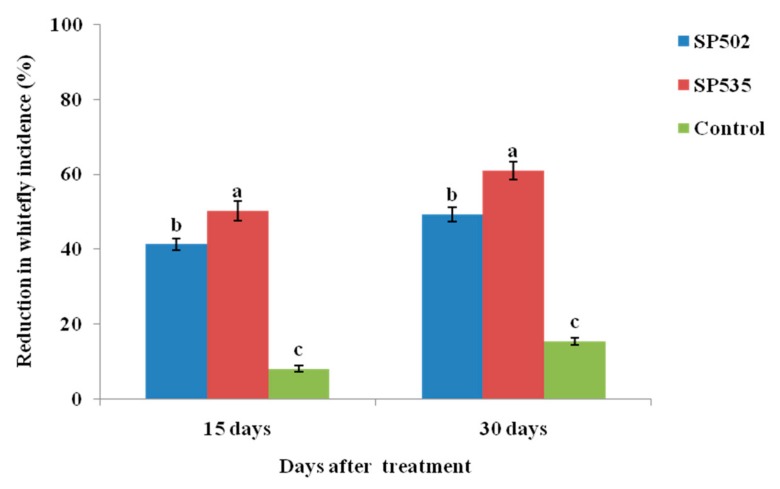
Reduction in whitefly incidence by plants colonized by different *Cordyceps fumosorosea* isolates after 15 and 30 days whitefly inoculation.

The colonization of eggplants by both *C. fumosorosea* isolates resulted in significant reduction of *B. tabaci* incidence when compared with control after 15 and 30 days of insect inoculation. The reduction in *B. tabaci* incidence in response to colonization by *C. fumosorosea* isolate SP535 was significantly higher than that observed for *C. fumosorosea* isolate SP502 (Figure 5).

## 4. Discussion

### 4.1. Effect of Fungal Inoculation on Plant Growth Parameters

Our results showed that plant growth and dry weight production was enhanced in response to seed treatment with *C. fumosorosea* (both isolates) when compared with control. These findings are consistent with the results of Jaber and Enkerli [8] who observed similar growth enhancement of broad bean when the seeds were treated with *B. bassiana* and *M. brunneum*. The variations among experimental replicates can be attributed to the number of biotic or abiotic factors. Our results are different from the findings of Akello et al. [33] who did not observe any change in the growth of banana plants treated with *B. bassiana,* whereas our results are at par with Gurulingappa et al. [34] and Lopez and Sword [35] who observed a significant increase in cotton and wheat growth in response to *B. bassiana* seed treatments. The above-mentioned inconsistencies of results can be related to the variations in the association of a particular fungal isolate with a plant species during the endophytic growth [10,36]. Our results are at par with Khan et al. [37] who observed a similar increase in plant dry weight of soybean in response to *Metarhizium anisopliae* treatment. The increase in plant dry weight can be explained by improvement in plant tolerance to herbivory and compensates for biomass lost due to insect attack [38,39].

### 4.2. Endophytic Colonization of Eggplant by C. fumosorosea

Overall both isolates of *C. fumosorosea* were found to endophytically colonize the root, stem, and leaves of eggplants. The extent or degree of fungal colonization was significantly similar during plant growth. Our results are similar to the findings of other researchers [8,36,40,41,42] who observed similar systemic plant colonization of endophytic fungi when fungi were applied as seed treatment. The decrease in percentage colonization with the passage of time may be caused by the responses of host plant to heterotrophic fungi, expansion of plant tissues with the maintenance of established colonies, or possible competition from other endophytes in the plant [43].

### 4.3. Effect of C. fumosorosea Colonization on Growth of B. tabaci

The colonization of eggplants by both isolates of *C. fumosorosea* caused a significant reduction in whitefly populations inoculated on plant leaves (after 15 and 30 days of whitefly inoculation) when compared with control. These results are similar to previous studies [2,33,34,44,45,46,47,48]. Based on the studies available to date, it has been well documented that endophytic entomopathogens can reduce the herbivore performance through production of secondary metabolites/compounds during the endophytic colonization of plants [49,50]. The reduction in insect populations on colonized plants can be related to the development stage of target pest or fungal species/strain being applied [47,51,52].

## 5. Conclusions

This study shows that seed treatment with *C. fumosorosea* solutions (two isolates) caused an increase in plant growth. *C. fumosorosea* isolate SP535 successfully colonized different plant parts and this colonization caused a reduction in whitefly populations on eggplant leaves. Our results provide basic information on enhanced plant growth and increased insect mortality in eggplants inoculated with two different isolates of *C. fumosorosea* in the absence of any imposed plant stress, which may be related to the possible production of secondary metabolites/compounds during the endophytic colonization of plants.

## Figures and Tables

**Table 1:** Effect of *C. fumosorosea* colonization of egg hatchability (%) of *B. tabaci.*

Treatments	Egg Hatchability (%) at Different Intervals Post Treatment	
	15 Days	30 Days
SP502	82.97 ± 2.01 c	80.34 ± 3.56 c
SP535	75.45 ± 3.94 b	73.10 ± 2.64 b
Control	92.30 ± 1.32 a	91.84 ± 1.23 a
*F*; *df*; *P*	23.41; 2, 12; <0.001	18.06; 2, 12; <0.001

Means in the same column followed by different letters are significantly different from each other at 5% level of significance. ± indicates the standard error of means based on five replicates (n = 5).

**Table 2:** Percentage mortality of *B. tabaci* pupa in response to colonization of *Cordyceps fumosorosea.*

Treatments	Pupal mortality (%) at Different Intervals Post Treatment	
	15 Days	30 Days
SP502	20.82 ± 2.01 b	28.40 ± 3.56b
SP535	25.97 ± 3.94 a	33.19 ± 2.64 a
Control	3.52 ± 1.32 c	4.21 ± 1.23 c
*F; df; P*	35.89;2,12;<0.001	43.25;2,12;<0.001

Means in the same column followed by different letters are significantly different from each other at 5% level of significance. ± indicates the standard error of means based on five replicates (n = 5).
